# Herbivore-Specific, Density-Dependent Induction of Plant Volatiles: Honest or “Cry Wolf” Signals?

**DOI:** 10.1371/journal.pone.0012161

**Published:** 2010-08-17

**Authors:** Kaori Shiojiri, Rika Ozawa, Soichi Kugimiya, Masayoshi Uefune, Michiel van Wijk, Maurice W. Sabelis, Junji Takabayashi

**Affiliations:** 1 Center for Ecological Research, Kyoto University, Shiga, Japan; 2 The Hakubi Center, Kyoto University, Yoshida, Kyoto, Japan; 3 National Institute for Agro-Environmental Sciences, Tsukuba, Japan; 4 Institute for Biodiversity and Ecosystem Dynamics, University of Amsterdam, Amsterdam, The Netherlands; University of Zurich, Switzerland

## Abstract

Plants release volatile chemicals upon attack by herbivorous arthropods. They do so commonly in a dose-dependent manner: the more herbivores, the more volatiles released. The volatiles attract predatory arthropods and the amount determines the probability of predator response. We show that seedlings of a cabbage variety (*Brassica oleracea var. capitata*, cv Shikidori) also show such a response to the density of cabbage white (*Pieris rapae*) larvae and attract more (naive) parasitoids (*Cotesia glomerata*) when there are more herbivores on the plant. However, when attacked by diamondback moth (*Plutella xylostella*) larvae, seedlings of the same variety (cv Shikidori) release volatiles, the total amount of which is high and constant and thus independent of caterpillar density, and naive parasitoids (*Cotesia vestalis*) of diamondback moth larvae fail to discriminate herbivore-rich from herbivore-poor plants. In contrast, seedlings of another cabbage variety of *B. oleracea* (*var. acephala*: kale) respond in a dose-dependent manner to the density of diamondback moth larvae and attract more parasitoids when there are more herbivores. Assuming these responses of the cabbage cultivars reflect behaviour of at least some genotypes of wild plants, we provide arguments why the behaviour of kale (*B. oleracea var acephala*) is best interpreted as an honest signaling strategy and that of cabbage cv Shikidori (*B. oleracea var capitata*) as a “cry wolf” signaling strategy, implying a conflict of interest between the plant and the enemies of its herbivores: the plant profits from being visited by the herbivore's enemies, but the latter would be better off by visiting other plants with more herbivores. If so, evolutionary theory on alarm signaling predicts consequences of major interest to students of plant protection, tritrophic systems and communication alike.

## Introduction

Plants release specific blends of volatile chemicals in response to insect herbivory, thereby attracting the herbivore's enemies [1]–[3]. In the cases studied so far, the composition of the herbivore-induced volatiles show little change with the number of herbivores inflicting damage to the plant, but the amounts of volatiles and the responses of the enemies increase significantly [4]–[6]. Signal quantity may therefore provide information to the enemies about herbivore abundance. In this respect, the plant signals an “honest” message. No (herbivore-induced) signal implies no herbivores and the more of the (herbivore-induced) signal, the more herbivores there are on the plant. It is too early to conclude that this is the general pattern, since the number of tritrophic systems studied is still small, but one may ask whether such a pattern, assuming it is common in the plant population, would be evolutionarily stable. The answer is negative [7], [8]; Any mutant plant that – even when herbivore-free or under attack of just a few herbivores – produces large amounts of volatiles, corresponding to herbivore-induced volatiles from common (resident) plant genotypes, would gain by attracting enemies, thereby receiving protection against herbivory for the duration of their visits. Predatory arthropods, true predators and parasitoids alike, cannot assess from a distance how many herbivores there are on a given plant. So, they have to rely on the chemical information. Thus, if most plants are ‘honest signalers’, the mutant plant is likely to attract predatory arthropods and thereby gain protection from herbivory, because upon arrival the predators will spend some time exploring the mutant plant for what it has to offer in terms of herbivores or alternative foods. At some point the predator may discover that the plant has little to offer and then leave, but unwillingly it has given a benefit to the mutant plant in terms of protection against any herbivore already present or attempting to settle. Such mutant plants overproducing the signal are further referred to as dishonest or “cry wolf” signalers, a term first used in the biological literature in relation to alarm calling behaviour of birds [8]. “Cry wolf” plants create a conflict of interests with the enemy of its enemies: the plant does not provide information to the herbivores' enemies about the number of potential prey it harbours [9]. Such conflicts are of paramount importance in driving the evolutionary dynamics of herbivore-induced plant signals: when plants sending honest signals are common, there is opportunity for mutants sending dishonest signals and once plants sending dishonest signals become too common, plants sending new-and-honest signals will be favoured by selection. Evolutionary models of the “cry wolf” game predict sustained waves of honest and dishonest signals under a broad range of conditions [10], [11].

We hypothesize that plant genotypes employing a “cry wolf” strategy are most successful in attracting predatory arthropods that have not yet learned to associate the signal (a complex blend of plant volatiles) with the reward (herbivore abundance) on the plant. Such plants may thus exploit the individuals in the predator population, that are endowed with some innate capacity to respond to quantitative or qualitative features of plant signals alone, yet naive as to how this chemical information exactly correlates with herbivore density. There is much evidence for innate responses in predatory arthropods to odours [12] and also to herbivore-induced plant volatiles [7], [13]–[16]. Thus, for a plant to deceive predators of its herbivores it should apply one of the following two strategies of releasing volatile chemicals: (1) herbivore-independent release (quality and quantity of odour remains constant), (2) release dependent on presence, but independent of abundance of herbivores (odour quality and quantity change due to presence, but not due to herbivore density on a plant). Whereas the first strategy is expected when physiological and ecological costs of releasing volatile chemicals are low compared to the benefits, the second strategy is expected when the cost-benefit balance switches sign after herbivore attack. Honest signaling plants are then defined as plants whose release is dependent on presence and abundance of herbivores (odour quality or odour quantity – or both – change with herbivore density per plant). As explained above, there is much evidence for this honest strategy, but – to our best knowledge – little or none for “cry wolf” strategies of the first type and certainly none for that of the second type. Even if none of these ideal types exist according to the artificial ‘nose’ (e.g. GC-MS) of a chemist, they may still effectively exist according to the ‘nose’ of the predators. In particular, naive predators may not be able to tell the difference even when odour quantity and/or odour quality changes with herbivore density. We aim to test plant signaling responses to different herbivore densities and to test the ability of predators of these herbivores to discriminate between the chemical information offered by the plant. To assess the plant signalling response directly (instead of only indirectly via the parasitoid's behavioural response), we also aim to identify and quantify the chemicals released from herbivore-infested plants.

In this article, we explore patterns of release of volatile chemicals in relation to the density of herbivores on seedlings of *B. oleracea* (cabbage or kale) and test for the response of specialist parasitoids of each of these herbivores. This study involved two herbivorous insects – larvae of cabbage white butterflies (CWB) and diamondback moths (DBM) – and two parasitoids – one gregarious species, *Cotesia glomerata*, specialized in Japan on CWB larvae only and the other a solitary species, *Cotesia vestalis*, specialized on DBM larvae. The herbivores impose differential threat to *Brassica* plants. CWB lays one or a few eggs per plant and avoids laying eggs on plants occupied by conspecifics [17]–[19], whereas DBM lays several eggs per plant per day (more than 100 over its life time) and prefers ovipositing on plants with conspecifics [20]–[22]. In addition, DBM concentrates its attack on young plant parts vital for plant growth. Given these contrasting risks, *Brassica* plants may take herbivore-specific countermeasures. We asked whether the release of herbivore-induced plant volatiles (HIPV) depends on herbivore species and, in particular, how the quantity and quality of released chemicals depends on herbivore abundance per plant. The experiments described in this article were designed to discriminate between the three above-described strategies of density-(in)dependent plant signal release, and to test the ability of naive parasitoid females to discriminate between the chemical information associated with different densities of caterpillars of their preferred host species on *B. oleracea* seedlings. The two caterpillar species on cabbage seedlings were thus used for independent trials to challenge the same plant for its response to herbivore density and the two parasitoid species were used to challenge their ability to estimate the density of their preferred host from the chemical information alone. Bio-assays were repeated with the same parasitoids and herbivore larvae, but on another variety of *B. oleracea*, to investigate whether signal release was controlled by the plant rather than the herbivore.

## Materials and Methods

### Plants and insects

Seedlings of cabbage plants (*Brassica oleracea* L. *var. capitata*, cv. Shikidori) were grown in a climate room for 4–5 weeks up to the 5-leaf stage (c. 15 cm high; including plant pot c. 20 cm high). Throughout this article, this cabbage variety was used except for one separate series of experiments (see two-choice tests), where another variety of *B. oleracea* (*var. acephala*: kale) was used for reasons of comparison.

Adult females of CWB, *Pieris rapae* (L.) (Lepidoptera: Pieridae), collected from a cabbage field in Kyoto City, in which various cultivars of *B. oleracea* were grown. They were allowed to lay eggs on cabbage plants (*B. oleracea var. capitata* cv Shikidori) in a cage. Upon hatching, CWB larvae were transferred to and reared on detached cabbage leaves (same cultivar). Young larvae of DBM, *Plutella xylostella* (L.) (Lepidoptera: Plutellidae) were collected from the field and reared in exactly the same way as CWB larvae.

Adult parasitoids of *Cotesia glomerata* and *C. vestalis* (Hymenoptera: Braconidae) were obtained from parasitized, field-collected larvae of CWB and DBM respectively. Cocoons of each parasitoid species were collected and kept in different glass tubes until emergence. To ensure mating, emerged females were put together with males in a plastic cage for 3 days. Thereafter, they were maintained in glass tubes at 18±2°C to prolong lifespan and in continuous darkness to suppress flight. Thus, the adult female parasitoids used for our experiments had no experience with plant volatiles and are therefore considered to be naive. They were maximally 10 days old since emergence from the host, had no foraging experience on herbivore-infested plants nor oviposition experience, and had 1–2 h acclimatization in the climate room before the experiments started.

### Two-choice tests

Female parasitoids of *C. vestalis* and *C. glomerata* were tested one-by-one for their flight response toward two potted seedlings of cabbage plants placed in an acryl cage (25×30×35 cm; 3 nylon-gauze-covered windows and one door) in a climate room at 25±2°C, 50–70% RH, continuous fluorescent light (20W, 3000 lux) without directed airflow [23]. They were released individually from a glass tube, positioned halfway the distance between the two potted cabbage seedlings. They repeatedly hovered over the plants inside the cage and, then, upon their first visit to a plant (defined as landing on the plant and subsequent initiation of ambulatory search), they were removed by an aspirator. The plant visited by the parasitoid was scored as their choice. Per replicate experiment usually 10 wasps were tested sequentially using the same set of two potted seedlings. Each treatment had 3 or more independent replicate experiments with new sets of parasitoids, treated plants and control plants. The number of parasitoids tested per replicate experiment was not always exactly 10, but varied from 8 to 23 depending on the conditions, the availability of wasps for testing and the time available per day.

Prior to each experiment, the two potted cabbage plants in the acryl cage had received different degrees of damage from either CWB or DBM. To prepare cabbage seedlings with 5, 15 and 30% damage, we placed respectively 1, 5 and 10 third-instar CWB larvae per plant and allowed feeding for 24 hours. Since DBM larvae feed less than CWB larvae, three times as many third-instar larvae of DBM (i.e. 3, 15 and 30) were allowed to feed per plant per day to obtain damage levels similar in amount and pattern to CWB. Two-choice tests were carried out for cabbage seedlings with the following contrasts in herbivore-inflicted damage: 5% *vs* 0%, 15% *vs* 0%, 15% *vs* 5%, 30% *vs* 5%, 30% *vs* 15%. These (as well as higher) damage levels per plant are realistic under field conditions in Japan (Uefune, pers. obs.). Prior to the tests, the larvae, their silk and their faeces were removed from the infested plants by the aid of a brush.

These two-choice tests were first carried out with the parasitoids *C. glomerata* and *C.vestalis* when offered the *B. oleracea* variety Shikidori, infested by CWB (for *C. glomerata*) or DBM (for *C. vestalis*) caterpillars. Next, to test for a role of the plant variety, the experiments were repeated with kale, *B. oleracea* variety *acephala*, using the same populations of DBM and the parasitoid *C. vestalis* (thus, no experiments with the parasitoid *C. glomerata* and CWB on this variety). Two-choice data were analyzed using replicated *G*-tests [24] (*H_o_*: parasitoids distribute equally over the two plants). Parasitoids that made no choice for either plant were discarded from this analysis.

### Analysis of leaf volatiles

Using Twister [polydimethylsiloxane (PDMS) coating stir bar, film thickness 0.5 mm, 10 mm length, Gerstel GmbH and Co. KG], volatile compounds were collected from the headspace of a single potted cabbage seedling (c. 15 cm high) that were inside 2 *l* glass bottles. Collection was done during daytime, shown previously to be the most relevant period for plant-parasitoid communication (parasitoids do not forage at night [23]). The collection was done for a period of 2 hrs, which sufficed to reach equilibrium adsorption on Twister. The plants were undamaged or damaged by either CWB caterpillars or DBM caterpillars. In addition, headspace collection was done from the headspace of mechanically damaged plants (*i.e.* 15% of total leaf area was damaged by punching holes), in order to assess which volatiles are induced by herbivores. For each treatment, headspace volatiles were collected three times, each time using a different plant (and, if required, new herbivores, fresh mechanical damage were applied following the same procedure as explained in the section on ‘two-choice tests’). These headspace collections were analyzed by GC-MS with an HP-5MS capillary column (Agilent Technologies, Inc.), equipped with thermo-desorption, cold-trap injector (Gerstel GmbH and Co. KG). GC-oven temperature was programmed to rise from 40°C (9 min. hold) to 280°C at 10°C/min. Compounds were identified if their mass spectra and retention times matched those in our own data base or those of synthetic compounds. Some compounds were mixtures of stereo-chemical isomers, but this was not further analysed.

We used a two-step procedure to test whether plant response (release of volatiles) increases with herbivore dose or alternatively stays constant and high, yet becomes only zero in absence of herbivory. First, we identified plant volatiles promoted after herbivory. This was done using a Student's *t*-test [24] for comparing mean ion intensities of each compound (or all compounds together) between undamaged plants (3 replicates) (or mechanically damaged plants (3 replicates)) and herbivore-damaged plants (all three damage levels together; hence, 3×3 = 9 replicates). If significantly different, the compound was considered to be herbivore-induced. If significantly different, the compound was considered to be herbivore-induced. The second step in the procedure then comprised of a regression of ion intensities on herbivore-damage level [24], thereby excluding the treatments without herbivores. This yielded estimates of intercept, slope and significance level of the regression of ion intensities (as a measure of emission) on damage level (which corresponds to a given number of larvae used to create this damage to the cabbage plant). For any given volatile compound, a significant and positive (negative) slope was interpreted as an increase (decrease) of its release with the damage level (and thus also with the number of herbivores creating this damage to the plant), whereas non-significance of the slope indicated that the release stays constant with the damage level (and thus also with the number of herbivores creating this damage to the plant).

### Synthetic volatiles

Assessing how the behavioural response of the parasitoids and the profile of volatile compounds changes with herbivore density on *B. oleracea* plants are two essential experiments to do, but determing which volatile compounds or mixtures thereof explain the response of the parasitoids is another. Because the behaviour of *C. vestalis* and the chemical profiles associated with DBM-infested *B. oleracea* plants showed the most striking and diverse features (see Results section), we decided to focus on the identification of compounds determining the response of the parasitoid *C. vestalis* to DBM-infested plants. To this end, only those pure synthetic compounds that corresponded to DBM-induced ones and the mixtures of these compounds only were offered to the parasitoids in two-choice tests. We aimed to assess which (combination of) compounds and which concentrations of these compounds maximize the response of the parasitoids, but – given the very large number of experiments required – we refrained from determining the quantitative blend composition that maximizes the parasitoid response. The synthetic compounds in pure form ((*Z*)-3-hexenyl acetate, *n*-heptanal, (+)-α-pinene, (–)-α-pinene, sabinene, *R*-(+)-limonene, *S*-(–)-limonene) (RC Treatt, Suffolk, UK; Wako Chemicals Co. Ltd., Osaka, Japan; Tokyo Kasei Kogyo Co. Ltd. Tokyo Japan) were dissolved in hexane (0.01 or 0.1 *mg* per compound per *ml* hexane) and a 10 *ml* solution was applied to a small cellulose sponge (2.5×2.5×0.5 cm). They were offered in amounts that – based on GC analysis – made their emission from the solution comparable to that from an infested cabbage plant (5% damage level). In case of stereochemical isomers, each was tested separately, whereas racemic mixtures were used in tests with mixtures of volatile chemicals. To achieve slow volatilization the sponge was enveloped by two sealed, polyethylene sacs (5×7 cm). GC-MS showed emission (ion intensities) per min (relative to control with 10 *ml* hexane) to approximate that from a cabbage plant with 5% DBM damage. In two-choice tests, odour and control sacs were positioned on the soil next to an uninfested cabbage seedling in a pot topped with a plastic cylinder (plant-high and pot-wide) to force odour release from the top of the cylinder. Thus, synthetic volatiles and their mixtures were always tested against a background of green leaf (i.e. intact plant) volatiles emanating from the uninfested cabbage seedling.

Using GC-analysis, ratios of compounds in synthetic odour mixtures were tuned to those from an infested cabbage plant. For the synthetic mixture of pure sabinene, *n*-heptanal, α-pinene and (*Z*)-3-hexenyl acetate, this was achieved with a ratio of 1.8:1.3:2.0:3.0.

Using triethyl citrate (TEC) as a solvent, a dilution series was prepared. A piece of filter paper (2×2 cm) in a dish was impregnated with 200 mg of TEC solution. The dish was placed on soil near the foot of a potted cabbage seedling. Using GC-MS, quantitative emission (ion intensities) from TEC-solutions per min was assessed and compared to that from pure TEC and a cabbage plant with 5% DBM damage.

To test whether the response of the parasitoids first increases and then decreases with the dose of synthetic volatile chemicals, the dose-response curve was split in two equal data sets (3 doses, each with 4 replicates of the response), left and right of the maximum response (*i.e.* between 10^−6^ and 10^−5^ dilution). Doses were log-transformed and responses per replicate (fraction positive) were arcsin(*√x*)-transformed. Each set was subject to regression analysis to test whether the slope of the regression line was significantly different from zero. The slopes of the regressions for the two halves of the data set were used to make inferences on the qualitative form of the dose-response relation: is there a maximum response at intermediate concentrations or not? We did not aim to assess the precise functional form.

## Results

### Parasitoid Flight preference to plants with differential herbivore damage

**Figure 1:**
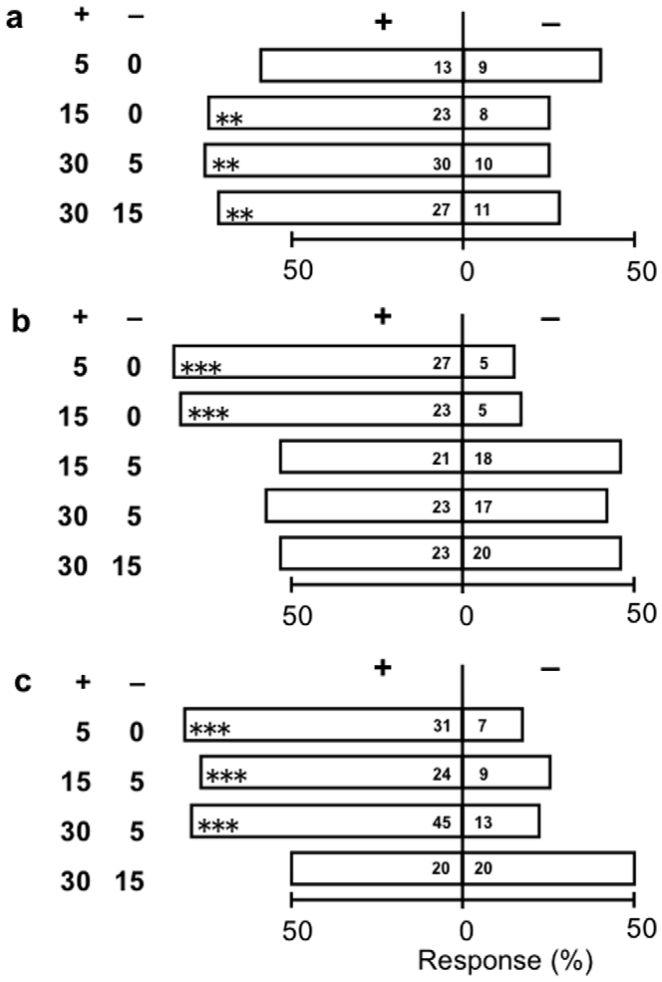
Two-choice tests for the response of parasitoid species to plants differing in herbivore damage: (1a) response of *Cotesia glomerata* to cabbage (*B. oleracea var. capitata* cv Shikidori) damaged by CWB larvae, (1b) response of *Cotesia vestalis* to cabbage plants (cv Shikidori) damaged by DBM larvae, and (1c) response of *Cotesia vestalis* to kale (*B. oleracea var acephala*) damaged by DBM larvae. Combinations of two plants (+, −) with different damage levels (0, 5, 15, 30%) are shown at the left-hand side of the vertical bars. Within each bar expressing response percentage, two numbers refer to totals of parasitoids, that visited respectively the + plant and the – plant offered in the test and asterisks refer to significance level according to replicated *G*-tests (* 0.01<*P*≤0.05; ** 0.001<*P*≤0.01; *** *P*≤0.001). The two-choice results per replicate experiment and the statistics of the replicated *G*-tests for (1a), (1b) and (1c) are shown respectively in Tables S1, S2 and S3.

When given a choice between two differentially damaged cabbage plants (cv Shikidori) (0, 5, 15 or 30% of their leaf surface damaged by CWB or DBM larvae, but larvae, silk and faeces removed prior to test), *C. glomerata* parasitoids landed preferentially on cabbage plants (cv Shikidori) that had more CWB damage (Figure 1a). This increasing dose-response relation is consistent with the literature to date (Turlings et al. 1995; Gols et al. 2003; Horiuchi et al. 2003). However, *C. vestalis* parasitoids showed a flat dose-response relation; they preferred to land on plants with DBM damage, yet did not discriminate between contrasting levels of plant damage (Figure 1b). Evidently, *C. vestalis* did not or could not determine whether cabbage plants harbour few or many DBM larvae, whereas *C. glomerata* preferred cabbage plants with more CWB larvae.

To test whether the latter phenomenon was unique for the plant, the two-choice tests were repeated with the same population of *C. vestalis* parasitoids and the same population of DBM caterpillars, but a different variety of *B. oleracea* (*var acephala*: kale). It was found that *C. vestalis* could discriminate between odours from kale seedlings with different levels of damage (i.e. 5% vs 0%, 15% vs 5%, 30% vs 5%, but not 30% vs 15%) (Figure 1c). This contrasts sharply with the responses to cabbage cv Shikidori (Figure 1b) and indicates that cabbage cv Shikidori may have some special properties.

Because parasitoids in all two-choice tests were removed immediately after first landing on a plant, they must have based their decision on distant sensory assessment. Vision alone is unlikely to explain the flat dose-response of *C. vestalis*. Hence, we focused on olfaction and carried out the GC-MS analysis decsribed below.

### Analysis of volatiles from plants with differential damage

**Figure 2:**
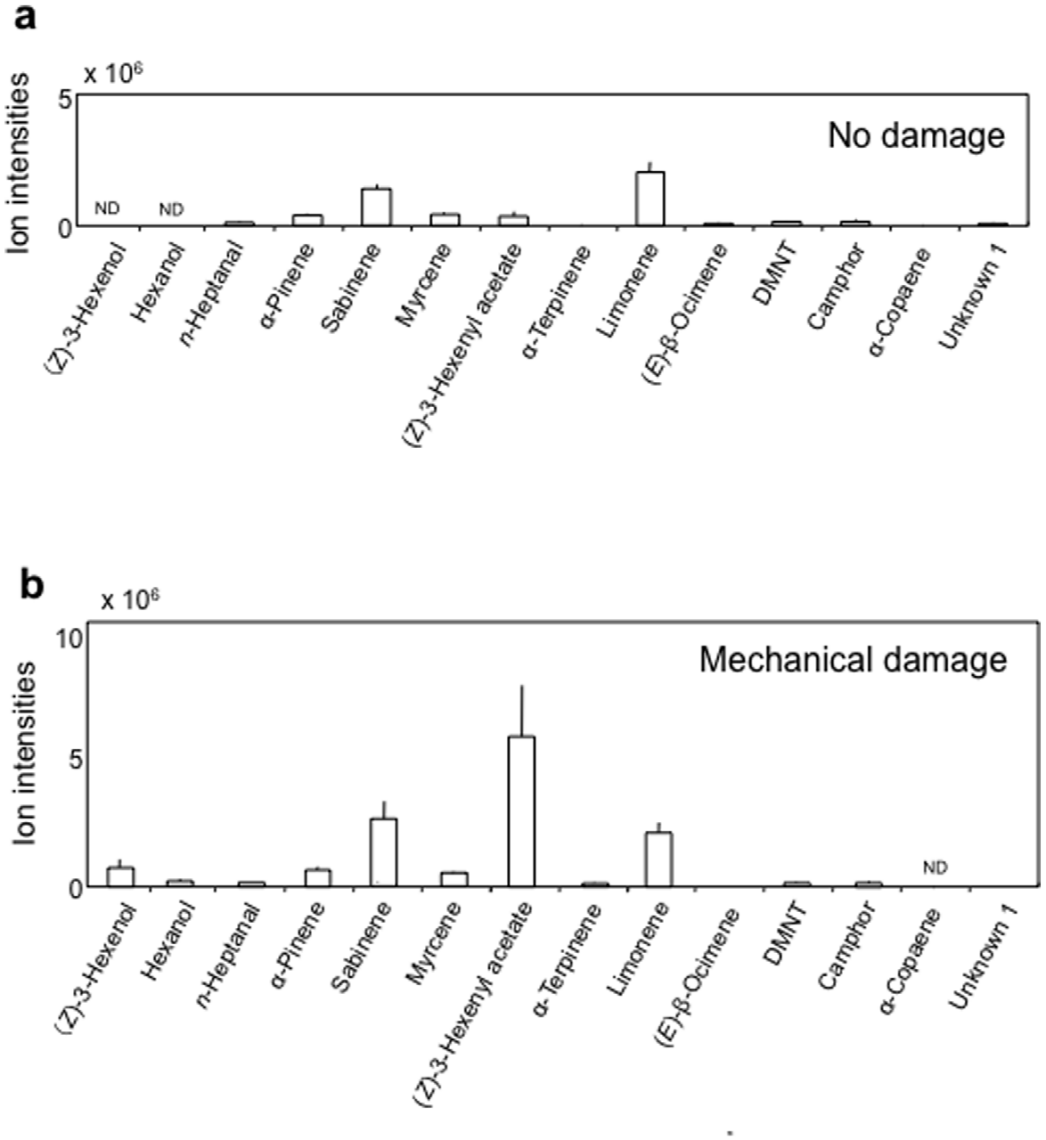
GC-MS analysis of headspace volatiles from herbivore-free cabbage (*B. oleracea var capitata* cv Shikidori). Plants had either no damage (2a), mechanical damage (2b).

**Figure 3:**
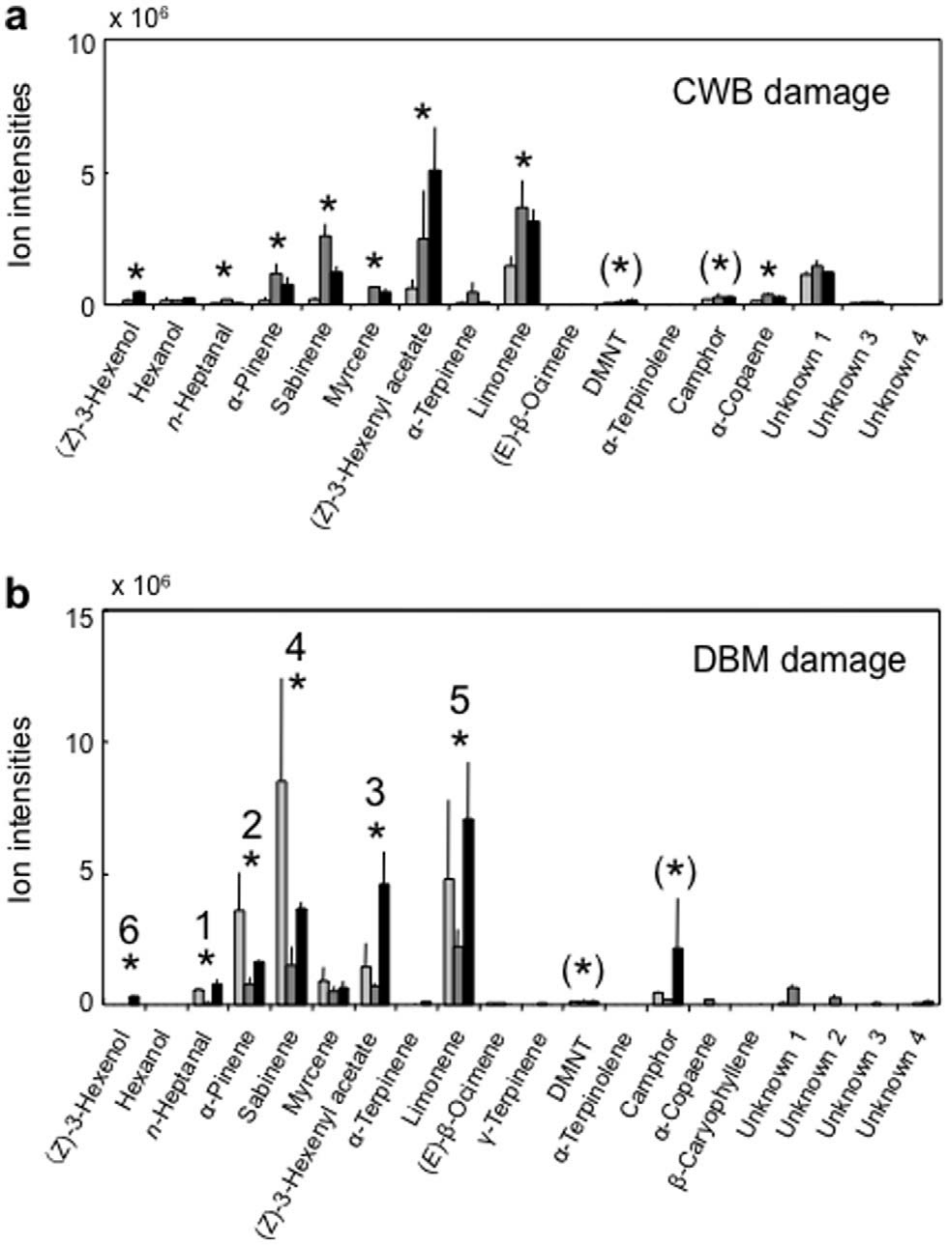
GC-MS analysis of headspace volatiles from herbivore-damaged cabbage (*B. oleracea var capitata* cv Shikidori). Plants had either 5, 15 or 30% damage by CWB larvae (3a) or by DBM larvae (3b). For each compound (*x*-axis), mean ion intensities (*y*-axis), plus standard error (vertical bars), are shown for emission from plants without damage (white column) and with either of three damage levels (in order of increasing damage: greyish, grey and black columns). DBM-induced volatiles are highlighted by superposed rank numbers according to significance level from Student's *t*-tests (these ranks serve as reference in Figure 4ab and 5).

We performed replicated GC-MS analyses to identify volatiles released from cabbage plants (cv Shikidori) that had ‘no damage’ (Figure 2a), mechanical damage (15% of leaf area was damaged by punching holes) (Figure 2b) or herbivore damage of various degrees (0, 5, 15 and 30%), yet with larvae, silk and faeces removed (as in two-choice tests) (Figure 3a, b). A Student's *t*-test for comparing mean ion intensities of volatiles from undamaged and herbivore-damaged plants – taking all non-zero damage levels together – revealed a significant increase in production of volatiles emanating from CWB-damaged plants (*P* = 0.014) and DBM-damaged plants (*P* = 0.009) (See also Table S5.) For CWB-damaged plants, 8-10 compounds increased in concentration (Figure 3a; according to Student's *t*-test significant for (*Z*)-3-hexenol, *n*-heptanal, α-pinene, sabinene, myrcene, (*Z*)-3-hexenyl acetate, limonene and α-copaene and bordering significance for camphor and DMNT, acronym for (*E*)-4,8-dimethyl-1,3,7-nonatriene; (Table S5); for DBM-damaged plants, 6-8 compounds increased in concentration (Figure 3b; according to Student's *t*-test significant for 6 compounds highlighted by rectangles and bordering significance for α-copaene and α-terpinolene; Table S5). (*Z*)-3-hexenol and (*Z*)-3-hexenyl acetate were induced by mechanical damage (i.e. punching holes in leaves), but also by herbivory, probably due to mechanical effects of larval mandibulae chewing leaves (Figure 2b and 3ab). The other HIPV probably emerge from biochemical interactions between plant and herbivore.

**Figure 4:**
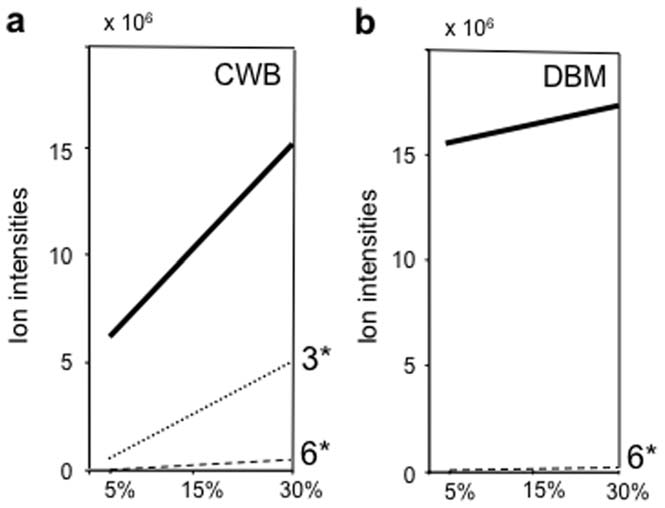
Regressions of total ion intensities on damage level. Drawn bold lines represent regressions of total ion intensities on damage level expressed in %, as in Figure 1 (corresponding to number of larvae/plant/day) for all volatiles from CWB-damaged (4a) and DBM-damaged (4b) cabbage plants (Table S4). For specific volatiles, only regressions with significant slope are additionally shown by dashed and dotted lines: for CWB (Figure 4a) (*Z*)-3-hexenyl acetate and (*Z*)-3-hexenol and for DBM (Figure 4b) only (*Z*)-3-hexenol.

Recall that the response of the parasitoid *C. glomerata* strongly depended on the density of CWB larvae on the cabbage plant (i.e. CWB-damage level). Strikingly in agreement with this density-dependent response, the quantity of all HIPV together increased significantly with damage level for CWB-damaged cabbage plants (cv Shikidori), whereas it stayed constant and high at all damage levels for DBM-damaged cabbage plants (cv Shikidori) (Figure 4a, b; Table S4). For CWB-damaged plants, each of the HIPV increased with damage level, but this was significant only for the two compounds induced by mechanical damage ((*Z*)-3-hexenol and (*Z*)-3-hexenyl acetate) and bordering significance for one compound (limonene) (Figure 4a). For DBM-damaged plants, the two compounds induced by mechanical damage increased with damage level, one significantly ((*Z*)-3-hexenol) and one bordering significance ((*Z*)-3-hexenyl acetate), but all other HIPV either increased or decreased non-significantly (Table S4). Moreover, the only significant regression on damage level, obtained for (*Z*)-3-hexenol, had a very small, positive slope, implying that the increase was quantitatively quite minor. Thus, the quantity of all CWB-induced cabbage volatiles together and two or perhaps three CWB-induced volatiles ((*Z*)-3-hexenol, (*Z*)-3-hexenyl acetate and limonene) provide information about CWB density. However, the quantity of DBM-induced cabbage volatiles does not provide information about DBM density nor do the specific volatiles. Perhaps, (*Z*)-3-hexenol is an exception, but this would require great sensory acuity for small quantitative changes (since the slope of the regression is so small and the absolute quantities are 4 times smaller than when CWB-induced). We conclude that there is variability in the volatiles induced in cabbage (cv Shikidori) by CWB larvae and in those by DBM larvae, but while the former do show significant quantitative relations with CWB density, the latter do not show such trends (or at best very little). Strikingly, the quantities of all DBM-induced volatiles together are always very high (even when compared with the highest quantities of volatiles induced by CWB in our experiments).

Using the same populations of DBM, GC-MS analysis was repeated for kale (*B. oleracea var. acephala*). Here, the quantities of all DBM-induced volatiles together increased significantly with herbivore density according to the regression statistics, shown only in Table S6. This shows that cabbage variety matters to the response induced by DBM larvae.

Given the insights in how the plant volatiles induced by the two herbivores differ with herbivore densities, we may now ask whether the parasitoids use information contained in these odour blends to assess herbivore density? The behavioural responses of the parasitoid *C. glomerata* depend on CWB density on the signal-releasing cabbage plant, but those of *C. vestalis* depend on presence not on density of DBM on the signal-releasing cabbage plant. The distinct responses of these two parasitoids show parallels with blend quantity released by the plant: the total amount of odour increases with CWB density, just as the response of the parasitoid *C. glomerata*, but it stays constant and high with DBM density, just as the response of the parasitoid *C. vestalis*. Qualitative changes in blend composition with CWB density (two or three compounds) may play a role for *C. glomerata*, but such changes with DBM density are unlikely to play a role for *C. vestalis*. Below, we analyse the response of *C. vestalis* to pure volatiles, mixtures of volatiles and total amount of volatiles in more detail.

### Flight preference of *C. vestalis* to pure and mixed synthetic chemicals

Using naive *C. vestalis*, the answer to the above question was sought by two-choice tests with each of the relevant chemicals alone or mixed in different concentrations against a background of odour from uninfested (clean) cabbage plants. Pure compounds dissolved in hexane were offered in amounts that – based on GC analysis – made their emission from the solution comparable to that from an infested cabbage plant (5% damage level). We selected (*Z*)-3-hexenyl acetate, *n*-heptanal, (+)-α-pinene, (–)-α-pinene, sabinene, *R*-(+)-limonene, *S*-(–)-limonene for the bio-assays because these compounds were found to increase significantly in response to herbivory. However, (*Z*)-3-hexenol was not selected because at the lowest DBM-damage level it was not significantly different from intact plants.

**Figure 5:**
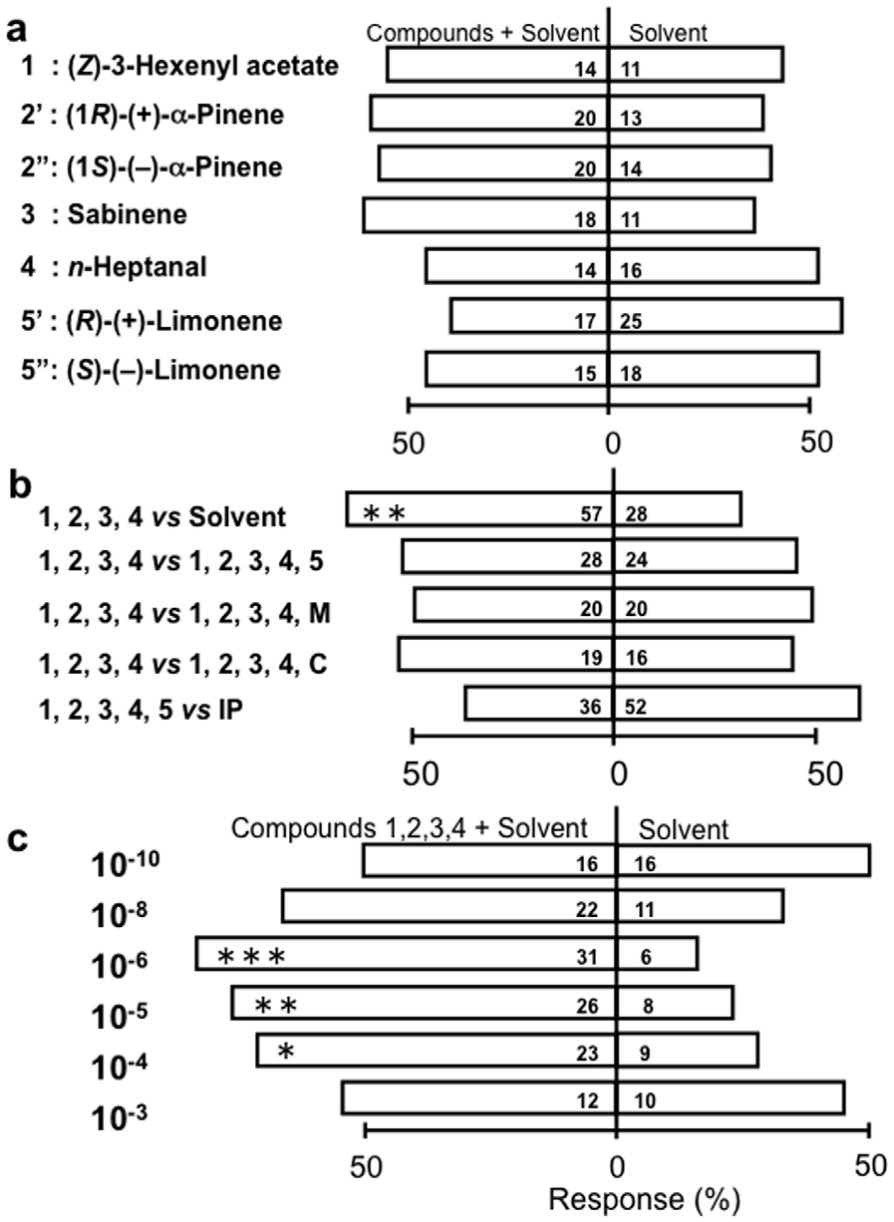
Response of parasitoid *Cotesia vestalis* to synthetic volatiles. Choice of parasitoid when offered (5a) pure, (5b) mixed (pure sabinene, *n*-heptanal, α-pinene and (*Z*)-3-hexenyl acetate in a ratio of 1.8:1.3:2.0:3.0) and (5c) a dilution series of mixed synthetic volatiles against the solvent (in 5abc) or the complete blend emitted from DBM-infested cabbage plants (only in 5b). Within each bar expressing response percentage, two numbers refer to totals of parasitoids, that visited respectively the + plant and the – plant offered in the test and asterisks refer to significance level according to replicated *G*-tests (* 0.01<P≤0.05; ** 0.001<P≤0.01; *** P≤0.001). Results per replicate and from replicated G-tests are shown in Tables S7, S8 and S9. Pure and mixed chemicals dissolved in hexane (0.01 *mg*/compound/*ml* hexane) (5a, 5b) or diluted in TEC (5c; dilution factor at left-hand side) are indicated by rank numbers attributed in Figure 3b or by capital letters, where M =  myrcene, C =  camphor represent two arbitrary additional compounds and the notation IP refers to a DBM-infested plant. Note that emission at 10^−6^ dilution in TEC approximates that from the hexane solution used in Figure 5b.

When offered alone against pure solvent, none of the pure compounds elicited a significant preference in naive females of the parasitoid (Figure 5a). However, when offered in mixtures against pure solvent, four compounds stand out as eliciting a significant preference (Figure 5b): *n*-heptanal, α-pinene, sabinene, (*Z*)-3-hexenyl acetate. This mixture did not become more attractive by adding myrcene, camphor or limonene, and was just not significantly different in attractiveness from DBM-induced cabbage odour (Figure 5b). Thus, – against a background of clean cabbage odours – mixtures of 4 HIPV triggered innate chemotaxis in naive parasitoids, whereas the individual compounds did not.

Presence-absence recording of the above 4 compounds would be the simplest mechanism explaining the flat response of *C. vestalis* to herbivore density. However, this mechanism cannot explain why this parasitoid responds differentially to blend quantity in a dilution series of the 4 compounds using triethyl citrate (TEC), as can be inferred from the parabolic shape of the dose-response relation (Figure 5c). Attraction was highest at a dilution factor of 10^−6^ and – according to regression analysis on data below and data above this maximum – decreased significantly toward higher doses (*P* = 0.013) as well as toward lower doses (*P* = 0.006). Thus, rather than presence-absence recording of compounds, the quantity of the odour compounds matters to the response of *C. vestalis*, a finding similar to what is recently shown for honeybees [25]. Taking ion intensities in the GC-MS analysis as a measure of quantity emitted, the 10^−6^ dilution in TEC was found to be equivalent to the solutions used in our experiments (0.01 *mg* of each compound per *ml* hexane), as well as equivalent to the DBM-induced cabbage odour. Thus, we conclude that cabbage plants (cv Shikidori) responded similarly to minor and severe attack in that they released a similar quantity of odour, and in that this quantity elicited a maximum response of naive *C. vestalis*.

## Discussion

### Density-dependent vs. density-independent plant responses

Commonly, plants release alarm signals upon attack by herbivorous insects and the total amounts released increase with herbivore density, thereby providing information to predators about the abundance of herbivores, their prey (see introduction). Our experiments reveal that cabbage seedlings also show such a positive response to the density of CWB caterpillars (Figure 4a): with an increase in CWB density, the total amount of all induced volatiles together increased significantly and the amounts of some induced volatiles increased, two significantly (Figure 4a) and one almost significantly (limonene, see Table S4). Apparently, these positively density-dependent changes in the amount of volatiles released enable naive females of the specialist parasitoid *C. glomerata* to discriminate between odours emanating from cabbage seedlings that harboured different numbers of CWB caterpillars as shown in the two-choice tests (Figure 1a). We therefore conclude that cabbage seedlings are honest signalers in their interaction with CWB caterpillars and naive females of the parasitoid *C. glomerata*.

However, when attacked by caterpillars of DBM, they release volatile chemicals, the amount of which is high, constant and thus independent of the number of caterpillars on the plant (Figure 4b). The composition of the blend showed variability, but no trend with the density of DBM caterpillars: in Figure 4b the only one compound that increased significantly ((*Z*)-3-Hexen-1-ol; Table S4) had a very small slope of the regression (so small that it would show up as an almost horizontal line in Figure 4b) and occurred in quantities 4 times lower than in CWB-induced blends (Supplemental information, Table S4). Moreover, our two-choice tests show that naive females of the specialist parasitoid *C. vestalis* cannot discriminate between odour blends from cabbage seedlings that had different numbers of DBM caterpillars (Figure 1b). We therefore conclude that cabbage seedlings are dishonest signalers in their interaction with DBM caterpillars and naive females of the parasitoid *C. vestalis*: they provide information to the parasitoid about the presence of DBM caterpillars, but provide evidently not enough information about their density. We interpret this result as first evidence for the effective existence of a “cry wolf” strategy of the second type described in the introduction.

### “Cry wolf” signalling: byproduct or strategy?

We have two reasons to interpret this phenomenon as a strategy of the plant to “cry wolf” and not as an accidental byproduct of the herbivores used in the experiments. One reason is that DBM caterpillars from the very same population induced density-independent ( = “cry wolf”) release of volatile attractants in one variety of *B. oleracea*, cv Shikidori, but DBM-density-dependent release in another variety of *B. oleracea* (*var acephala*: kale). The other reason is that the DBM caterpillars would not benefit from making the plant “cry wolf” because the effect is that the plant lures more parasitoids. Admittedly, conclusive proof can come only from analyzing the plant-molecular mechanisms underlying recognition and response to its attackers. The point in case is that cabbage seedlings attacked by just a few DBM caterpillars are induced to “cry wolf” whether this is intentional or just a by-product of the organisms used. This plant behaviour creates a conflict of interest between the plant and the herbivore's enemies. The plant's interest is to receive protection against herbivory by luring the herbivore's enemies as bodyguards. This interest is largely independent of the number of herbivores it harbours and, hence, plants may “cry wolf”. However, the herbivore's enemies would be better off by visiting plants with many caterpillars (for example: *C. vestalis* can parasitize c. 6 caterpillars per patch per hour (Uefune, per. obs.), but cannot discriminate between cabbage plants harbouring 3, 15 and 30 caterpillars). Yet, they cannot avoid being attracted to “cry wolf” plants, because their sensory equipment does not enable them to assess from a distance how many herbivores there are on the plant. Moreover, they cannot avoid spending time on a “cry wolf” plant, because they need time to acquire information about herbivore abundance by exploring the plant surface by ambulatory search. In this way, the plant receives protection against herbivores for the time spent by parasitoids exploring its surface for potential hosts.

Whereas the term “cry wolf” strategy implies an act of deceit (i.e. ‘plant deceits the enemies of the plant’s attackers), it is not self-evident that it evolved for that reason. Deceit may well be a by-product of the plant's strategy to lure the herbivore's enemies in advance of the aggregated herbivore attack that will take place after initial attack. As explained in the introduction, DBM tends to aggregate on plants already under DBM attack. Thus, even when DBM larvae come in small numbers initially, the plant is likely to incur aggregated attack in the future. By attracting the herbivore's enemies at an early stage of herbivore attack the plant may reduce or even suppress the build-up of a herbivore aggregation in two ways: (1) by killing herbivores and (2) by scaring them off. This ‘early attract’ hypothesis may not only explain why cabbage plants respond to DBM attack in a density-independent way, but it may also explain why they do not do that in response to CWB attack. This is because CWB does not aggregate on cabbage plants under CWB attack: CWB females avoid ovipositing on CWB-infested plants. However, note that the ‘early attract’ hypothesis does not explain why the other *Brassica* variety (kale) shows a density-dependent increase in the production of DBM-induced volatiles.

Thus, high and density-independent HIPV release may well be or may not be deceitful to the parasitoids attracted. The crucial question is whether the interests of the parasitoid line up with the interests of the plant. If they do, we have no reason to invoke the “cry wolf” interpretation', but, if not, there is room for this interpretation. We argue below that the interests of the cabbage cultivar and the parasitoid under investigation are not at all likely to match. For the parasitoid female arriving on a cabbage plant with few DBM caterpillars it does not pay to wait for the DBM aggregation to develop in the future. This is because the life span of the parasitoid *C. vestalis* under favourable conditions (ample honey available) is shorter [26] than the time required for the aggregative oviposition response of DBM to result in caterpillar stages suitable for the parasitoid (i.e. 2^nd^ and 3^rd^ instars; [27]–[29], i.e. at least 2 weeks [30]. Moreover, an individual female of the parasitoid *C. vestalis* may tend not to deposit all its eggs in one patch to avoid inbreeding (resulting in diploid, infertile males under one-locus complementary sex determination [31], [32]) and hence she will spread her eggs to some extent over different DBM aggregations. Thus, a parasitoid, like *C. vestalis*, may not gain by staying on the plant for many days and waiting for DBM to aggregate. As a consequence, the interests of the parasitoid and the cabbage plant are not likely to match and the cabbage plant can only gain by luring as many of these parasitoids as they can, possibly by deceit.

So, how does the plant manage to deceive the parasitoid? Recall that the parasitoids in our experiments were naive: they had no prior experience with HIPV, plant and (density of) hosts. Evidently, these naive females of the parasitoid *C. vestalis* did not extract more information from the qualitative differences in DBM-induced odours from cabbage plants with different numbers of DBM caterpillars. Would they have had a learning experience, they might have learned to discriminate between relevant blend qualities. Here, we did not test the role of learning, because it is not needed to make our main point. For this article it suffices to argue that the environment is likely to harbour parasitoids, and predators alike, that are naive with respect to subtle qualitative differences in HIPV (i.e. signal quality) and the number of herbivores on the plant (i.e. the reward). It is exactly the presence of this reservoir of naive parasitoids and predators that creates the opportunity for plants that play a “cry wolf” strategy. Whether plants also manage to play this strategy against parasitoids and predators that had time to learn, is a question beyond the scope of this article and thus remains an open question for future research.

We do not claim to have shown the role of “cry wolf” strategies in plant populations, but only to have detected the basic ingredients of such a strategy in seedlings of a cabbage variety. To reveal how it works in the field, still requires a considerable amount of work. Given that our experiments were carried out in small cages in the laboratory, one would like to see evidence from experiments in the field. However, we have substantial evidence from field experiments that the blend of 4 volatiles and their amounts play a decisive role in the attraction of *C. vestalis* to cabbage plants and to the parasitisation rates of DBM caterpillars (M. Uefune, unpublished data) and that the range of DBM abundances per plant matches that observed on cabbage plants in the field, as well as on at least some related wild plants in Japan (*Rorippa indica*, *Brassica juncea*) (J. Abe and M. Uefune, pers obs).

### “Cry wolf” strategies of what type?

In the introduction we distinguished two types of “cry wolf” strategies. To the best of our knowledge our results are the first to show any correspondance with “cry wolf” strategies of the second type: one variety of cabbage plants reveal herbivore presence, but not their abundance. However, the case of molasses grass (also called stinkgrass; *Melinus minutiflorus*) in Kenia might represent a case of “cry wolf” strategies of the first type [33]. The constitutive release of odours by this grass causes stem borers to avoid utilizing this plant as a food source and – most strikingly – causes females of the parasitoid *Cotesia sesamiae* to frequent this plant. These effects of molasses grass on stem borer avoidance and parasitoid attraction appear to be so strong that intercropping this plant in stands of maize causes the maize to suffer less from stem borer damage and to harbour a larger percentage of *Cotesia*-parasitized stem borers [33]. It would be highly interesting to test whether herbivore infestation alters the blends released by molasses grass and whether the parasitoids attracted were naive with respect to herbivores on this grass. Moreover, one may ask whether stem borers avoid molasses grass because of its direct defences and/or because of the indirect defences accomplished by luring parasitoids that in turn act as plant bodyguards.

We hypothesize that plants may even go a step further than shown in this article. If they offer alternative foods (*e.g.* nectar, pollen) in addition to signals, they could motivate enemies of herbivores to stay and thereby acquire protection for an even longer time [34], [35]. In this way, “cry wolf” plants may gain protection by exploiting the initial naivety of the herbivore's enemies and by arresting them upon arrival by plant foods as a surrogate for herbivores as food.

### “Cry wolf” strategy as a plant response to a specific herbivore

It is very interesting to observe that different herbivore species (CWB and DBM) induce a different response in the same plant cultivar (cv Shikidori) and that the same herbivore species (DBM) induce a different response in two different varieties (cultivars) of *B. oleracea* (*var capitata* cv Shikidori and *var. acephala*, kale). While this phenomenon was known to exist with respect to blend composition [36], our results also show that it exists with respect to shape of the plant response to herbivore density. This prompts new questions such as (1) how different herbivore species can induce responses that differ in whether they are dependent on herbivore density or not, (2) how plants somehow perceive for which herbivore species it is more important to cheat and to release high levels of volatiles irrespective of its density. While the energetic costs of producing of volatiles are probably low, the ecological costs of being conspicuous are likely to be high (e.g. [9]) and this may favour plant genotypes that tune their herbivore-induced response to the herbivore species they are attacked by.

### Population-level consequences of “cry wolf” plants

The detection of a “cry wolf” strategy in a plant cultivar is an important finding because in natural populations such strategies could drive a co-evolutionary game with selection acting on the herbivore's enemies to learn which plant signals are more profitable, and acting on plants to provide signals that are honest or dishonest with respect to how many herbivores they harbour. When honest signals are common, plant mutants sending dishonest signals will initially gain (less damage, equal attractiveness) and therefore increase in frequency. When dishonest signals are common, plant mutants sending honest signals may benefit from herbivore's enemies that learn to discriminate and frequent them (see also [10] for another argument emerging from spatial pattern formation and requiring no ability to learn). For a more simple version of the “cry wolf” game, i.e. without trophic levels, it has been shown that – in a large part of the parameter domain – frequency-dependent selection can drive sustained waves of individuals sending dishonest signals followed by others sending different, yet honest, signals, and so on [10], [11]. Such cycles are likely to be a feature of “cry wolf” models with trophic levels (Sabelis and van Baalen, unpublished). As a byproduct of the diversity in honest and dishonest signalers, there will be heterogeneity in attack rate per herbivore, which is known to promote the persistence of herbivores and their enemies [37]–[42]. Thus, we predict “cry-wolf” and “honest” signaling to coexist in natural plant populations, a hypothesis that requires an experimental test. Now that plant metabolic engineering of attractants has become feasible [43], [44], it may be worthwhile to understand the population-level consequences, before moving on to apply these molecular tools to agriculture in an attempt to increase crop attractiveness to the enemies of their pests.

## Supporting Information

Table S1 Replicated G-tests for two-choice experiments with the parasitoid *Cotesia glomerata* (Figure 1a), when offered two cabbage plants (cv Shikidori) that differ in the number of CWB larvae, feeding on them for one day. Asterisks refer to significance level. n = number of parasitoids landing on (+), (−) or no (0) source; GH, GP and GT are the values of the G-statistic for heterogeneity, pooled data and total; df  =  degrees of freedom; CWB  =  Cabbage white butterfly.
(0.03 MB DOC)

Table S2 Replicated G-tests for two-choice experiments with the parasitoid *Cotesia vestalis* (Figure 1b), when offered two cabbage plants (cv Shikidori) that differ in the number of DBM larvae, feeding on them for one day. DBM  =  Diamondback moth.
(0.03 MB DOC)

Table S3 Replicated G-tests for two-choice experiments with the parasitoid *Cotesia vestalis* (Figure 1c), when offered two cabbage plants (Kale) that differ in the number of DBM larvae, feeding on them for one day.
(0.03 MB DOC)

Table S4 Regression of GC-MS data (ion intensities) for volatiles emanating from (a) CWB-damaged and (b) DBM-damaged cabbage plants (cv Shikidori) on damage-level (# larvae/plant). Intercept, slope and standard error (SE) are given in units of 103; Correlation coefficient (−1<r<+1), significance level (P), non-significance (NS).
(0.03 MB DOC)

Table S5 Student's t-tests on GC-MS data to compare volatile chemicals from herbivore-free cabbage plants (cv Shikidori) with those infested by different numbers of CWB or DBM larvae.
(0.03 MB DOC)

Table S6 Regression of GC-MS data (ion intensities) for total of volatiles emanating from DBM-damaged cabbage plants (Kale) on damage-level (# larvae/plant). Intercept, slope and standard error (SE) are given in units of 103; Correlation coefficient (−1<r<+1), significance level (P), non-significance (NS).
(0.03 MB DOC)

Table S7 Replicated G-tests for two-choice experiments with the parasitoid *Cotesia vestalis* (Figure 5a) when offered volatile chemicals (1, 2, 3, 4, 5, as in Figure 3b, or each of their stereochemical isomers) in hexane solution (+) against pure solvent (−).
(0.03 MB DOC)

Table S8 Replicated G-tests for two-choice experiments with *Cotesia vestalis* (Figure 5b) when offered synthetic blends of four, five or six volatile chemicals in a hexane solution (compounds 1, 2 (racemic mix), 3, 4, 5 (racemic mix) as in Figure 3b and Table S7; M  =  Myrcene, C  =  Camphor) or natural blends from DBM-infested plants (IP).
(0.03 MB DOC)

Table S9 Replicated G-tests for two-choice experiments with *Cotesia vestalis* (Figure 5c) when offered blends of four volatile chemicals (1, 2, 3 and 4, as in Figure 3b) in a tri-ethyl citrate solution at different dilutions.
(0.03 MB DOC)
