# Supplementary material for: Herbivore-Specific, Density-Dependent Induction of Plant Volatiles: Honest or “Cry Wolf” Signals?
Source: PLoS One. 2010 Aug 17;5(8):e12161. doi: 10.1371/journal.pone.0012161 (PMC2923144; doi:10.1371/journal.pone.0012161)
Supplement: Table S1 — Replicated G-tests for two-choice experiments with the parasitoid Cotesia glomerata (Figure 1a), when offered two cabbage plants (cv Shikidori) that differ in the number of CWB larvae, feeding on them for one day. Asterisks refer to significance level. n = number of parasitoids landing on (+), (−) or no (0) source; GH, GP and GT are the values of the G-statistic for heterogeneity, pooled data and total; df = degrees of freedom; CWB = Cabbage white butterfly. (0.03 MB DOC) [file pone.0012161.s001.doc]

**Table S1 Replicated G-tests for two-choice experiments with the parasitoid *Cotesia glomerata* (Figure 1a), when offered two cabbage plants (cv Shikidori) that differ in the number of CWB larvae, feeding on them for one day. Asterisks refer to significance level. n = number of parasitoids landing on (+), (–) or no (0) source; G_H_, G_P_ and G_T_ are the values of the G-statistic for heterogeneity, pooled data and total; df = degrees of freedom; CWB = Cabbage white butterfly.**

# CWB larvae

*(+) (–) n(+) n(–) n(0) G_H_(df) G_P_(df) G_T_(df)*

1 0 2 2 6 3.172 (3) *NS* 0.731 (1) *NS* 3.903 (4) *NS*

2 1 3

5 4 7

4 2 9

5 0 8 3 0 0.354 (2) *NS* 7.572 (1)** 7.926 (3)*

10 4 0

5 1 2

10 1 15 5 0 0.000 (1) *NS* 10.465(1)** 10.465(2)**

15 5 0

10 5 13 7 0 0.760 (1) *NS* 6.951 (1)** 7.712(2)*

14 4 0

*P* = Significance level; NS P>0.10; BS 0.05<P≤0.10; * 0.01<P≤0.05; ** 0.001<P≤0.01; *** P≤0.001
