# Supplementary material for: Herbivore-Specific, Density-Dependent Induction of Plant Volatiles: Honest or “Cry Wolf” Signals?
Source: PLoS One. 2010 Aug 17;5(8):e12161. doi: 10.1371/journal.pone.0012161 (PMC2923144; doi:10.1371/journal.pone.0012161)
Supplement: Table S2 — Replicated G-tests for two-choice experiments with the parasitoid Cotesia vestalis (Figure 1b), when offered two cabbage plants (cv Shikidori) that differ in the number of DBM larvae, feeding on them for one day. DBM = Diamondback moth. (0.03 MB DOC) [file pone.0012161.s002.doc]

**Table S2 Replicated *G*-tests for two-choice experiments with the parasitoid *Cotesia vestalis* (Figure 1b), when offered two cabbage plants (cv Shikidori) that differ in the number of DBM larvae, feeding on them for one day. DBM = Diamondback moth.**

# DBM larvae

*(+) (–) n(+) n(–) n(0) G_H_(df) G_P_(df) G_T_(df)*

3 0 8 0 2 3.464 (3) *NS* 16.624 (1)*** 20.088 (4)***

6 2 0

6 1 1

7 2 0

15 0 7 2 0 0.186 (3) *NS* 12.540 (1)*** 12.726 (4)* 6 1 0

5 1 4

5 1 0

15 3 4 6 0 1.929 (3) *NS* 0.231 (1) *NS* 2.160 (4) *NS*

5 4 1

5 5 0

7 3 0

30 3 4 6 0 1.950 (3) *NS* 0.903 (1 *NS* 2.854 (4) *NS*

7 3 0

6 4 0

6 4 0

30 15 13 9 1 0.569 (1) *NS* 0.209 (1) *NS* 0.779 (2) *NS*

10 11 2

*P* = Significance level; NS P>0.10; BS 0.05<P≤0.10; * 0.01<P≤0.05; ** 0.001<P≤0.01; *** P≤0.001
