# Supplementary material for: Herbivore-Specific, Density-Dependent Induction of Plant Volatiles: Honest or “Cry Wolf” Signals?
Source: PLoS One. 2010 Aug 17;5(8):e12161. doi: 10.1371/journal.pone.0012161 (PMC2923144; doi:10.1371/journal.pone.0012161)
Supplement: Table S3 — Replicated G-tests for two-choice experiments with the parasitoid Cotesia vestalis (Figure 1c), when offered two cabbage plants (Kale) that differ in the number of DBM larvae, feeding on them for one day. (0.03 MB DOC) [file pone.0012161.s003.doc]

**Table S3 Replicated *G*-tests for two-choice experiments with the parasitoid *Cotesia vestalis* (Figure 1c), when offered two cabbage plants (Kale) that differ in the number of DBM larvae, feeding on them for one day.**

# DBM larvae

*(+) (–) n(+) n(–) n(0) G_H_(df) G_P_(df) G_T_(df)*

3 0 10 0 0 5.843 (3) *NS* 16.373 (1)*** 22.216 (4)***

7 1 2

7 3 0

7 3 0

15 3 4 2 4 2.012 (3) *NS* 7.075** (1) 9.086*BS* (4)

6 4 0

7 1 2

7 2 1

30 3 10 0 0 6.065 (5) *NS* 22.752*** (1) 28.818*** (6)

7 3 0

7 3 0

7 3 0

6 2 2

8 2 0

30 15 5 5 0 0.569 (3) *NS* 0.000 (1) *NS* 0.569 (4) *NS*

4 6 0

7 3 0

4 6 0

*P* = Significance level; NS P>0.10; BS 0.05<P≤0.10; * 0.01<P≤0.05; ** 0.001<P≤0.01; *** P≤0.001
