# Supplementary material for: Herbivore-Specific, Density-Dependent Induction of Plant Volatiles: Honest or “Cry Wolf” Signals?
Source: PLoS One. 2010 Aug 17;5(8):e12161. doi: 10.1371/journal.pone.0012161 (PMC2923144; doi:10.1371/journal.pone.0012161)
Supplement: Table S4 — Regression of GC-MS data (ion intensities) for volatiles emanating from (a) CWB-damaged and (b) DBM-damaged cabbage plants (cv Shikidori) on damage-level (# larvae/plant). Intercept, slope and standard error (SE) are given in units of 103; Correlation coefficient (−1<r<+1), significance level (P), non-significance (NS). (0.03 MB DOC) [file pone.0012161.s004.doc]

Table S4 Regression of GC-MS data (ion intensities) for volatiles emanating from (a) CWB-damaged and (b) DBM-damaged cabbage plants (cv Shikidori) on damage-level (# larvae/plant). Intercept, slope and standard error (SE) are given in units of 10^3^; Correlation coefficient (–1<*r*<+1), significance level (*P*), non-significance (NS).

**2a. CWB damaged plants**

Chemicals *Intercept(SE)* *Slope(SE)* *r* *P*

(Z)-3-Hexen-1-ol –78.8(88.5) +54.01(13.66) +0.831 0.006 **

n-Heptanal +81.1(37.7) +2.14(5.82) +0.138 0.724 *NS*

α-Pinene +368.9(354.1) +61.0(54.6) +0.389 0.301 *NS*

Sabinene +802.6(675.8) +99.1(104.3) +0.338 0.374 *NS*

Myrcene +116.0(166.3) +46.3(25.6) +0.564 0.114 *NS*

(Z)-3-Hexenyl acetate +53.4(1370.0) +499.2(211.4) +0.665 0.050 *

Limonene +1682.0(730.9) +231.0(112.8) +0.612 0.080 *BS*

DMNT +29.5(62.8) +14.01(9.69) +0.479 0.121 *NS*

Camphor +193.5(74.1) +11.23(114.4) +0.348 0.359 *NS*

α-Copaene +178.3(77.8) +15.22(120.0) +0.432 0.245 *NS*

All compounds +5191.0(2790.2) +998.2(430.5) +0.659 0.045 *

**2b DBM damaged plants**

(Z)-3-Hexen-1-ol –84.15(44.93) +11.55(2.31) +0.884 0.002 **

n-Heptanal +375.7(229.5) +7.29(11.80) +0.227 0.556 *NS*

α-Pinene +3073.9(1009.1) –66.5(51.9) –0.436 0.241 *NS*

Sabinene +7237.9(2653.7) –165.9(136.5) –0.417 0.264 *NS*

(Z)-3-Hexenyl acetate +323.1(1063.0) +121.3(54.7) +0.643 0.062 *BS*

Limonene +3191.7(2317.6) +94.0(119.2) +0.286 0.456 *NS*

DMNT +130.7(59.1) –0.34(3.04) –0.042 0.914 *NS*

α-Terpinolene +5.44(5.59) –0.04(0.29) –0.052 0.895 *NS*

α-Copaene +60.55(53.49) –0.24(2.75) –0.033 0.933 *NS*

All compounds +15478.4(7235.8) +61.7(372.2) +0.063 0.873 *NS*
