# Supplementary material for: Herbivore-Specific, Density-Dependent Induction of Plant Volatiles: Honest or “Cry Wolf” Signals?
Source: PLoS One. 2010 Aug 17;5(8):e12161. doi: 10.1371/journal.pone.0012161 (PMC2923144; doi:10.1371/journal.pone.0012161)
Supplement: Table S5 — Student's t-tests on GC-MS data to compare volatile chemicals from herbivore-free cabbage plants (cv Shikidori) with those infested by different numbers of CWB or DBM larvae. (0.03 MB DOC) [file pone.0012161.s005.doc]

Table S5 Student’s t-tests on GC-MS data to compare volatile chemicals from herbivore-free cabbage plants (cv Shikidori) with those infested by different numbers of CWB or DBM larvae

**Chemicals CWB damaged plants DBM damaged plants**

*P* (*t*-test ) *P* (*t*-test)

(Z)-3-Hexen-1-ol 0.015* 0.042*

1-Hexanol 0.396NS 0.173NS

n-Heptanal 0.013* 0.010**

α-Pinene 0.011* 0.013*

Sabinene 0.003** 0.038*

Myrcene 0.001*** 0.119NS

(Z)-3-Hexenyl acetate 0.026* 0.017*

α-Terpinene 0.164NS 0.096BS

Limonene 0.010** 0.040*

β-Ocimene 0.104NS 0.215NS

γ-Terpinene not detected 0.112NS

DMNT 0.060BS 0.244NS

α-Terpinolene 0.487NS 0.072BS

Camphor 0.061BS 0.127NS

α-Copaene 0.007** 0.065BS

β-Caryophyllene not detected 0.145NS

Unknown 1-5 0.155-0.346NS 0.085-0.173NS

Total 0.014* 0.009**

Note that DMNT = (E)-4,8-dimethyl-1,3,7-nonatriene (C11 homoterpene)

*P* = Significance level; NS P>0.10; BS 0.05<P≤0.10; * 0.01<P≤0.05; ** 0.001<P≤0.01; *** P≤0.001
