# Supplementary material for: Herbivore-Specific, Density-Dependent Induction of Plant Volatiles: Honest or “Cry Wolf” Signals?
Source: PLoS One. 2010 Aug 17;5(8):e12161. doi: 10.1371/journal.pone.0012161 (PMC2923144; doi:10.1371/journal.pone.0012161)
Supplement: Table S6 — Regression of GC-MS data (ion intensities) for total of volatiles emanating from DBM-damaged cabbage plants (Kale) on damage-level (# larvae/plant). Intercept, slope and standard error (SE) are given in units of 103; Correlation coefficient (−1<r<+1), significance level (P), non-significance (NS). (0.03 MB DOC) [file pone.0012161.s006.doc]

Table S6 Regression of GC-MS data (ion intensities) for total of volatiles emanating from DBM-damaged cabbage plants (Kale) on damage-level (# larvae/plant). Intercept, slope and standard error (SE) are given in units of 10^3^; Correlation coefficient (–1<*r*<+1), significance level (*P*), non-significance (NS).

Chemicals *Intercept(SE)* *Slope(SE)* *r* *P*

Total 17585.1(4911.3) +2357.7(720.8) +0.589 0.039*
