# Supplementary material for: Herbivore-Specific, Density-Dependent Induction of Plant Volatiles: Honest or “Cry Wolf” Signals?
Source: PLoS One. 2010 Aug 17;5(8):e12161. doi: 10.1371/journal.pone.0012161 (PMC2923144; doi:10.1371/journal.pone.0012161)
Supplement: Table S7 — Replicated G-tests for two-choice experiments with the parasitoid Cotesia vestalis (Figure 5a) when offered volatile chemicals (1, 2, 3, 4, 5, as in Figure 3b, or each of their stereochemical isomers) in hexane solution (+) against pure solvent (−). (0.03 MB DOC) [file pone.0012161.s007.doc]

**Table S7 Replicated G-tests for two-choice experiments with the parasitoid *Cotesia vestalis* (Figure 5a) when offered volatile chemicals (1, 2, 3, 4, 5, as in Figure 3b, or each of their stereochemical isomers) in hexane solution (+) against pure solvent (–).**

Chemicals *(μg/μl*) *n(+) n(–) n(0) G_H_(df) G_P_(df) G_T_(df)*

1: *n*-heptanal (0.01) 5 1 4 6.462 (3) 0.133 (1) 6.595 (4)

1 4 4

5 4 1

3 7 1

(+)-α-Pinene (0.01) 2 1 7 1.303 (3) 1.496 (1) 2.799 (4)

10 7 0

4 1 5

4 4 3

2: (+)-α-Pinene (0.1) 7 3 5 2.436 (3) 0.893 (1) 3.329 (4)

3 4 8

11 6 6

10 11 2

(–)-α-Pinene (0.01) 5 5 0 2.001 (3) 1.064 (1) 3.065 (4)

8 3 3

5 3 4

2 3 4

2’: (–)-α-Pinene (0.1) 3 2 1 3.987 (3) 1.397 (1) 5.384 (4)

3 6 2

1 6 3

3 2 5

3: (Z)-3-Hexenyl 1 2 7 1.200 (3) 0.361 (1) 1.561 (4)

acetate (0.01) 4 4 2

3 2 5

6 3 3

4: Sabinene (0.01) 5 5 2 1.692 (3) 1.706 (1) 3.399 (4)

5 2 2

4 1 3

4 3 1

5’: R-(+)-Limonene 5 8 7 1.139 (2) 1.533 (1) 2.672(3)

4 9 7

8 8 4

5’’: S-(–)-Limonene 3 6 11 1.436 (2) 0.273 (1) 1.709 (3)

6 4 10

6 8 6
