# Supplementary material for: Herbivore-Specific, Density-Dependent Induction of Plant Volatiles: Honest or “Cry Wolf” Signals?
Source: PLoS One. 2010 Aug 17;5(8):e12161. doi: 10.1371/journal.pone.0012161 (PMC2923144; doi:10.1371/journal.pone.0012161)
Supplement: Table S8 — Replicated G-tests for two-choice experiments with Cotesia vestalis (Figure 5b) when offered synthetic blends of four, five or six volatile chemicals in a hexane solution (compounds 1, 2 (racemic mix), 3, 4, 5 (racemic mix) as in Figure 3b and Table S7; M = Myrcene, C = Camphor) or natural blends from DBM-infested plants (IP). (0.03 MB DOC) [file pone.0012161.s008.doc]

Table S8 Replicated G-tests for two-choice experiments with *Cotesia vestalis* (Figure 5b) when offered synthetic blends of four, five or six volatile chemicals in a hexane solution (compounds 1, 2 (racemic mix), 3, 4, 5 (racemic mix) as in Figure 3b and Table S7; M = Myrcene, C = Camphor) or natural blends from DBM-infested plants (IP).

Blends (+ *vs* –) *n(+) n(–) n(0) G_H_(df) G_P_(df) G_T_(df)*

1,2,3,4 *vs* Solvent 30 16 12 1.189 (2) 10.096 (1)** 11.285 (3)*

11 7 3

16 5 8

1,2,3,4 *vs* 1,2,3,4,M 11 9 0 0.407 (1) 0.000 (1) 0.407 (2)

9 11 0

1,2,3,4 *vs* 1,2,3,4,C 8 9 3 0.698 (1) 0.257 (1) 0.955 (2)

11 7 2

1,2,3,4 *vs* 1,2,3,4,5 10 8 2 0.994 (2) 0.308 (1) 1.302 (3)

8 11 1

6 9 5

1,2,3,4,5 *vs* IP 9 10 1 1.933 (3) 2.925 (1) 4.858 (3)

6 12 2

6 13 1

15 17 3
