# Supplementary material for: Herbivore-Specific, Density-Dependent Induction of Plant Volatiles: Honest or “Cry Wolf” Signals?
Source: PLoS One. 2010 Aug 17;5(8):e12161. doi: 10.1371/journal.pone.0012161 (PMC2923144; doi:10.1371/journal.pone.0012161)
Supplement: Table S9 — Replicated G-tests for two-choice experiments with Cotesia vestalis (Figure 5c) when offered blends of four volatile chemicals (1, 2, 3 and 4, as in Figure 3b) in a tri-ethyl citrate solution at different dilutions. (0.03 MB DOC) [file pone.0012161.s009.doc]

Table S9 Replicated G-tests for two-choice experiments with *Cotesia vestalis* (Figure 5c) when offered blends of four volatile chemicals (1, 2, 3 and 4, as in Figure 3b) in a tri-ethyl citrate solution at different dilutions.

Dilution factor *n(+) n(–) n(0) G_H_(df) G_P_(df) G_T_(df)*

10^-10^ 6 2 2 6.038 (3) 0.030 (1) 6.069 (4)

1 4 5

6 4 0

3 7 0

10^-8^ 8 2 0 1.951(3) 3.738 (1) 5.688 (4)

4 4 2

5 3 2

5 2 3

10^-6^ 7 2 1 0.699 (3) 18.493*** (1) 19.192*** (4)

8 1 1

8 2 0

8 1 1

10^-5^ 6 2 2 1.704 (3) 10.033** (1) 11.738* (4)

8 1 1

5 3 2

7 2 1

10^-4^ 7 1 2 1.169 (3) 5.624* (1) 6.793 (4)

5 2 3

7 3 0

4 3 3

10^-3^ 3 2 5 4.858 (3) 0.182 (1) 5.040 (4)

5 1 4

3 3 4

1 4 5
